# Lingering Chukchi Sea sea ice and Chukchi Sea mean winds influence population age structure of euphausiids (krill) found in the bowhead whale feeding hotspot near Pt. Barrow, Alaska

**DOI:** 10.1371/journal.pone.0254418

**Published:** 2021-07-12

**Authors:** Carin J. Ashjian, Stephen R. Okkonen, Robert G. Campbell, Philip Alatalo

**Affiliations:** 1 Department of Biology, Woods Hole Oceanographic Institution, Woods Hole, Massachusetts, United States of America; 2 College of Ocean and Fisheries Science, University of Alaska Fairbanks, Fairbanks, Alaska, United States of America; 3 Graduate School of Oceanography, University of Rhode Island, Narragansett, Rhode Island United States of America; University of Connecticut, UNITED STATES

## Abstract

Interannual variability in euphausiid (krill) abundance and population structure and associations of those measures with environmental drivers were investigated in an 11-year study conducted in late August–early September 2005–2015 in offshelf waters (bottom depth > 40 m) in Barrow Canyon and the Beaufort Sea just downstream of Distributed Biological Observatory site 5 (DBO5) near Pt. Barrow, Alaska. Statistically-significant positive correlations were observed among krill population structure (proportion of juveniles and adults), the volume of Late Season Melt Water (LMW), and late-spring Chukchi Sea sea ice extent. High proportions of juvenile and adult krill were seen in years with larger volumes of LMW and greater spring sea ice extents (2006, 2009, 2012–2014) while the converse, high proportions of furcilia, were seen in years with smaller volumes of LMW and lower spring sea ice extent (2005, 2007, 2010, 2011, 2015). These different life stage, sea ice and water mass regimes represent integrated advective responses to mean fall and/or spring Chukchi Sea winds, driven by prevailing atmospheric pressure distributions in the two sets of years. In years with high proportions of juveniles and adults, late-spring and preceding-fall winds were weak and variable while in years with high proportions of furcilia, late-spring and preceding-fall winds were strong, easterly and consistent. The interaction of krill life history with yearly differences in the northward transports of krill and water masses along with sea ice retreat determines the population structure of late-summer krill populations in the DBO5 region near Pt. Barrow. Years with higher proportions of mature krill may provide larger prey to the Pt. Barrow area bowhead whale prey hotspot. The characteristics of prey near Pt. Barrow is dependent on krill abundance and size, large-scale environmental forcing, and interannual variability in recruitment success of krill in the Bering Sea.

## Introduction

The region near Pt. Barrow, Alaska is a known recurrent feeding region for bowhead whales as they migrate south in fall from the Canadian Arctic to overwinter south of Bering Strait [1, 2]. Here, euphausiids are frequently the dominant prey item consumed by the bowhead whales [3, 4]. The physical and biological factors that generate a favorable feeding environment for bowhead whales near Pt. Barrow were explored during an 11-year oceanographic study [5]. It was found that dense aggregations of bowhead whale prey, euphausiids or krill, on the shelf northeast of Pt. Barrow during the fall are formed through a wind sequence in which local wind-driven shelf-break upwelling that delivers krill to the shelf is followed by relaxation that results in krill being trapped on the shelf between westward-flowing shelf currents and the northeastward-flowing Alaskan Coastal Current in Barrow Canyon (the “krill trap”) [6–8]. A reliable supply of krill for the whales on the shelf at Pt. Barrow in fall depends not only on the occurrence of this sequence of winds but also on the characteristics of the krill (abundance, size) in the waters offshore.

Two species of euphausiids (herein krill) are common near Pt. Barrow: *Thysanoessa inermis* and *T*. *raschii*. A third species, *T*. *longipes*, is observed infrequently in low abundance. All are believed to be endemic to the Bering Sea and the Sea of Okhotsk and are present near Pt. Barrow after northward advection through the Chukchi Sea [9–11]. Although their habitats overlap, *T*. *inermis* is typically found on the outer shelf and the slope of the Bering Sea while the distribution of *T*. *raschii* is primarily on the inner-middle shelf [12, 13]. Both spawn during the spring (*T*. *raschii* in April-May; *T*. *inermis* in early April) and follow a 2–3 year multi-stage life cycle, spending the most time as furcilia and juveniles before reaching the adult stage [10]. Juveniles and adults are larger (~6–20 mm total length) than furcilia (~3–5 mm total length for late furcilia stages), and thus should provide larger prey for bowhead whales [10, 13]. Studies of development rate are rare, however *T*. *inermis* development from egg to furcilia stage 3 was estimated at 31–40 days at 7–10°C in the North Sea [14] and from egg to early-mid furcilia stages has been estimated at 30–40 days [14, 15] at warmer temperatures (5–10°C) in the northern Bering and Chukchi Seas during April and May (<1°C; [16–18]). Thus, krill spawned in the northern Bering Sea and Gulf of Anadyr that enter the Chukchi Sea may have only achieved early furcilia stages before passing through Bering Strait and should have 1–2 additional years of development before reaching the adult reproductive stages. Krill may overwinter in the Chukchi Sea, perhaps utilizing the under-ice or benthic environments.

**Fig 1:**
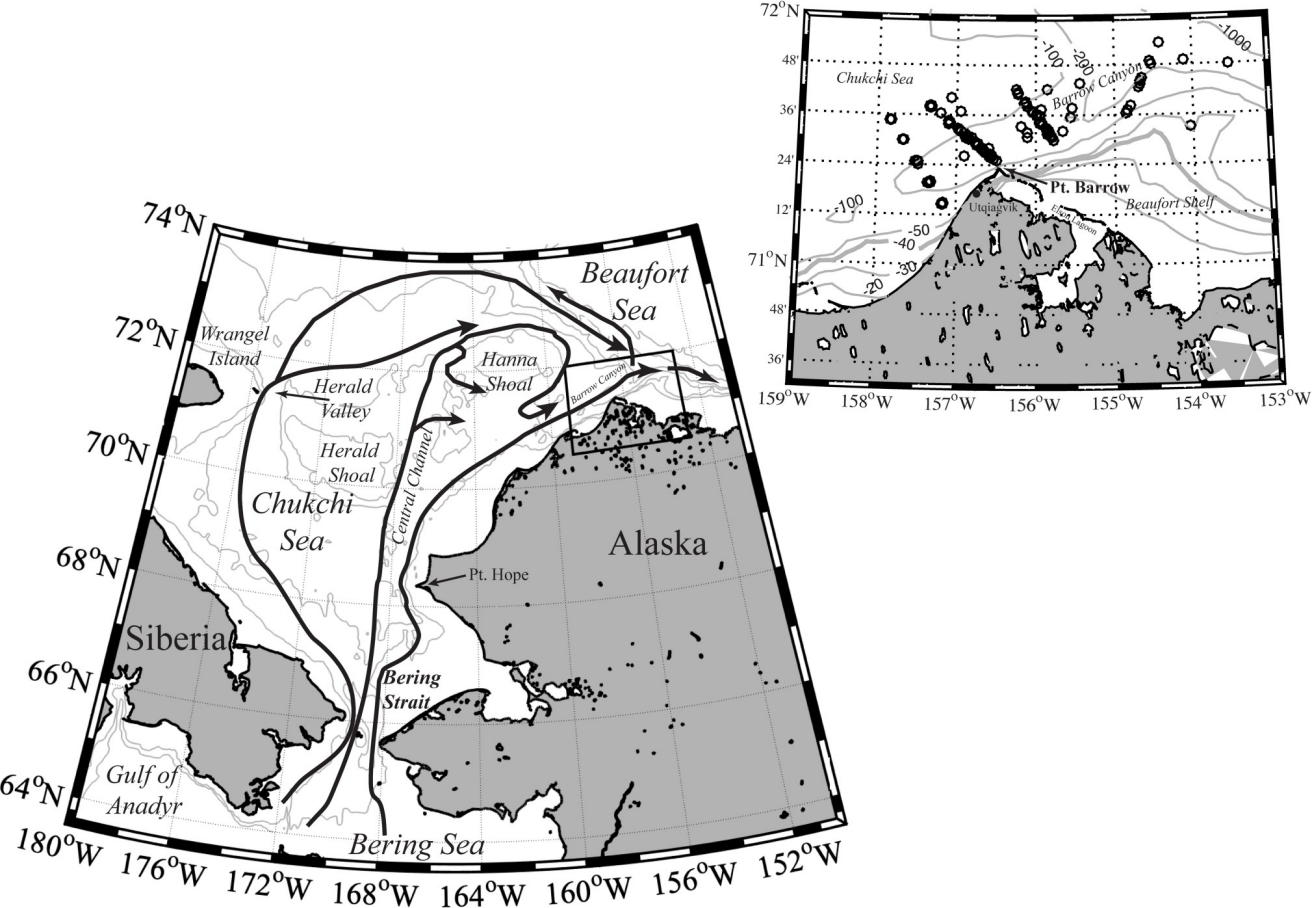
Geographic setting and dominant pathways of northward flow from the Bering Sea through the Chukchi Sea to Pt. Barrow, AK. Inset shows sampling locations near Pt. Barrow. Pathways of currents after *Brugler et al*. [19] and *Danielson et al*. [21]; not all currents are shown. Box indicates area enlarged in right panel. Many locations were sampled more than once and thus overlie each other. The 40-m isobath is denoted by the thicker gray line. Maps generated using Matlab/vR2016b.

Three dominant northward current pathways carry Pacific-origin water and plankton, including krill, through the Chukchi Sea (Fig 1): An eastern pathway associated with the Alaskan Coastal Current, a central Chukchi Sea pathway that runs through the Central Channel and then east around the southern and northern flanks of Hanna Shoal, and a western pathway that runs through Herald Valley and then eastward along the Chukchi slope [19–24]. Krill originating in the northern Bering Sea during spring can reach Pt. Barrow by fall through the eastern pathway but the central and western pathways require additional time for the transit [11, 18]. Krill entering through the western and central pathways likely originated in the Anadyr Gulf and the western Bering Sea while those entering through the eastern pathway may have originated on the Bering Sea shelf, with *T*. *raschii* originating on the inner-middle shelf and *T*. *inermis* on the outer shelf. Of particular relevance to this study, *Berline et al*. [11] identified two peak krill arrival events at Pt. Barrow; a fall peak inferred to be comprised of furcilia spawned earlier in the spring of the year and a subsequent peak the following year comprised of juveniles and adults (JAD), that is, furcilia that had survived and matured beneath the Chukchi ice canopy during the intervening winter and spring.

Chukchi Sea currents, water mass distributions, and sea ice extent and phenology are closely tied to atmospheric drivers. *Okkonen et al*. [5] demonstrated that Pacific Water and melt water volumes in Barrow Canyon were correlated with spring sea ice extent in the eastern Chukchi Sea and that interannual variability in the timing of sea ice retreat was governed by seasonal-averaged regional winds and the position of the Beaufort atmospheric high-pressure cell. Monthly and annual transports through Bering Strait are correlated with regional wind forcing and are, on an annual basis, increasing with time in response to greater forcing by southerly winds [17]. In response to the increased Bering Strait transport, the flushing time of the Chukchi Sea decreased from ~7.5 months in 2001 to ~4.5 months in 2014 [17], which has significant impacts on the residence time of krill populations in the Chukchi Sea.

In addition to providing a reliable feeding hotspot in fall for migrating bowhead whales, the region near Pt. Barrow lies at the confluence of Chukchi, Beaufort, and Arctic biomes, a site of elevated primary production that sustains a rich and abundant benthos, supports seabird, fish, and marine mammal populations, and is undergoing rapid environmental changes in response to changing climate [25–29]. Because of its ecological importance and ongoing environmental transformations, this region is included in the Distributed Biological Observatory (DBO) network, a latitudinal network of oceanographic transects that are sampled repeatedly over multiple months and years by international researchers, as DBO Site 5 [26, 30].

The eleven-year time series of oceanographic and zooplankton data from fall 2005–2015 [5] was collected in the DBO 5 region and provides an opportunity to further investigate some of the factors influencing the availability of krill near Pt. Barrow. Earlier work described the mechanism (krill trap) forming the krill hotspot near Pt. Barrow [6], associations between winds, krill presence on the shelf, as inferred from diel vertical migration (DVM) patterns in acoustic backscatter, and bowhead whales [7, 8], and atmospheric drivers of late summer water masses [5], but did not focus on the population structure and species composition of the krill themselves. Here the relationships between regional physical drivers and krill life stage characteristics and species composition in Barrow Canyon and over the Beaufort slope, the source for the krill on the shelf, are explored for an 11-year period. The governing paradigm of the analysis is that population structure (life stages) of the krill observed offshore near Pt. Barrow, and available for bowhead whales, are a consequence of prior *time-averaged wind-driven* circulation and of upstream conditions in the Bering Sea where the krill originate. Specifically, are there interannual differences in krill population structure at Pt. Barrow in late summer that are correlated with large-scale atmospheric drivers and associated changes in water masses and sea ice?

## Data and methods

### Oceanographic fieldwork

Oceanographic sampling was conducted as part of a multiple project effort during August-September, 2005–2015, near Pt. Barrow AK using the either the 50’ *R/V Ukpik* (2013) or the 37’ *R/V Annika Marie* (all other years) at stations (Fig 1) usually located along transects extending from the near shore across the shelf to the slope of Barrow Canyon. Only stations in water deeper than 40 m were considered in this analysis since those locations would contain the source populations of the krill (vs. the shallow shelf that would contain krill that had been upwelled and aggregated by the “krill trap” mechanism, potentially differentially by life stage or species). Hydrographic measurements were collected using an SBE 19plus Conductivity-Temperature-Depth (CTD) recorder (see *Okkonen et al*. [5, 31] for additional information). Samples for plankton abundance and composition were collected using either a 60-cm diameter, 5:1 single or double (Seagear Inc.) ring net equipped with 150 μm (or 200 μm, 2009 only) mesh and a flow meter (all years) and a 60-cm ¼ m^2^ Tucker trawl equipped with flow meter and two 350 μm mesh nets that sampled different depth intervals (2011–2015 only). Oblique tows were conducted at ~1–1.5 knots (ring net) or ~3 knots (Tucker) ship speed from the surface to a target depth of 5-m off of the seafloor or to as deep as the hydrowire permitted when in deeper water (usually ~150 m). Tows were conducted without regard to day or night, however all tows were conducted during daylight hours in 2009–2015 and 60–100% of tows were conducted during daylight hours or twilight (within one hour of sunset) in 2005–2008 (Table 1). Sampling depths were quantified using a Vemco Inc., Wildco Inc., or Star-Oddi Inc. time-depth recorder mounted on each net. The volume of water sampled was estimated from the flow meter counts or, for the Tucker trawl, the distance traveled by the net. Samples were preserved immediately after collection in 5% formalin seawater.

**Table 1:** Net tow information.

Year	Sampling Dates (m/d)	# Tows	# Tows with Krill	Day # (%)	Twilight # (%)	Night # (%)	% Day or Twilight
2005	8/21–9/8	11	11	7 (64)	1 (9)	3 (27)	73
2006	8/24–8/29	5	5	3 (60)	2 (40)	0 (0)	100
2007	8/22–9/7	14	13	10 (71)	0 (0)	4 (29)	71
2008	8/21–9/6	15	15	10 (67)	2 (13)	3 (20)	75
2009	8/25–8/26	7	7	7 (100)	0 (0)	0 (0)	100
2010	8/21–9/10	18	17	18(100)	0 (0)	0 (0)	100
2011	8/22–9/3	17 (19)	17 (19)	17 (94)	2 (18)	0 (0)	100
2012	8/29–9/9	11 (11)	11 (11)	9 (82)	2 (18)	0 (0)	100
2013	8/20–9/1	14 (15)	14 (14)	13 (93)	1 (7)	0 (0)	100
2014	8/21–9/5	14 (12)	13 (12)	14 (100)	0 (0)	0 (0)	100
2015	8/19–9/2	14 (15)	14 (15)	14 (100)	0 (0)	0 (0)	100
	Total Samples	141 (71)	136 (70)	122	10	10	92

For each year, the sampling dates (month/year), numbers of ring net and Tucker (in parentheses) tows from Barrow Canyon and the Beaufort Sea shelf-break and slope (bottom depths greater than 40), the number tows of each type that contained krill, the number and percentage of tows conducted during day, twilight, or night, and the percentage of tows conducted during day or twilight combined are shown. Twilight is defined as within one hour of sunset/sunrise.

### Zooplankton

Upon return to the laboratory, species and life stage composition and abundance were enumerated using successive splits of a plankton splitter to until at least 150 of several target organisms, including krill calyptopes, furcilia, juveniles, and adults, had been identified. For large taxa such as euphausiids/krill, the entire sample might be examined. Total or integrated abundance (#/m^2^) was determined using the concentration for each net (#/m^3^) and the measured depth of the two; for the two Tucker trawl nets, integrated abundance was calculated prior to summing the net abundances.

The relatively small mouth area, slow-towed ring net is not optimal methodology for quantitatively collecting relatively fast swimming, visually-capable krill, since net escapement was possible. Smaller nets were used because of limited deck space on the small research vessels. The ring net tows were conducted as part of an eleven-year effort to study mesozooplankton in this region rather than being targeted solely at krill. During the last 5 years of the project, the Tucker trawl also was used to better collect krill, however the longest data record (11 years) was obtained using the ring net. To mitigate the abundance underestimates, analyses addressing the research objectives of this study use the proportion of life stages in each sample from the ring net tows rather than the actual abundances. These proportions were compared between the Tucker trawl and ring net data to verify that the ring net data appropriately represented the metrics.

Mean abundances of krill for each year were calculated using all tows. The proportions of 1) combined juvenile and adult (JAD) krill, herein the JAD proportion and a rough measure of krill population structure and 2) each species in the total population (furcilia, juveniles, and adults) were calculated for all stations where krill were collected. Differences in these proportions between gear types were tested using the Wilcoxon Paired Sample T-Test [32]. Differences between years for each of the nets in total abundance and of the proportions of the difference species and of JAD were tested using the Kruskal-Wallis test followed by a non-parametric Tukey-Kramer type post-hoc test [32]. Pearson correlation coefficients were calculated between the JAD proportion and the total abundances for each net with significance levels from *Zar* [32].

### Sea ice

Daily sea ice concentrations were obtained from the NOAA High-resolution Blended Analysis of Daily SST and Ice dataset (https://www.esrl.noaa.gov/psd/data/gridded/data.noaa.oisst.v2.highres.html; [33]) at ¼° resolution. After correcting for anomalous concentrations, daily Chukchi Sea ice areas were computed (see [5] for details).

### Comparisons of krill population structure, sea ice concentration, water mass volumes, and Bering Strait transports

Comparisons were conducted using normalized anomalies from the 11-year mean. The anomaly from the mean (*a_i_*) was calculated as:

$$a_{i}=(x_{i}-X)$$ (1)

where *x_i_* is the observed value for year *i* and *X* is the mean of all values. The anomalies for each year then were normalized to the maximum of the absolute values of all of the years (Eq 2), where *N* is the number of years, generating normalized anomalies (*na_i_*) between -1 and 1.


$${na}_{i}=a_{i}∕max|a_{i=1,N}|$$ (2)

Late-spring eastern Chukchi Sea ice extent was represented here by anomalies from the 11-year mean sea ice concentration on May 31^st^ of each year; May 31^st^ was identified through iterative correlation analysis as having the highest correlation between sea ice and the proportion JAD (p<0.01); see *Okkonen et al*. [5] for more information on the approach). Volumes of Late Season Melt Water (LMW), Alaskan Coastal Water (ACW), Chukchi Summer Water (CSW), and Winter Water (WW) were calculated for each year in *Okkonen et al*. [5]. In brief, temperature and salinity data for each year from a sentinel transect across Barrow Canyon sampled in late August were interpolated to a common grid (1-km horizontal by 1-m vertical; 1000 m^3^/grid cell). The water mass present in each grid cell was identified based on T/S characteristics, following water mass definitions in *Okkonen et al*. [5]. Grid cell volumes for each water mass in each sampling year then were summed. Those values were here converted to normalized anomalies as discussed above. Pearson correlation coefficients were calculated between normalized anomalies with significance values from *Zar* [32].

### Meteorology

Daily sea level wind and sea level pressure (SLP) reanalysis products over the Bering-Chukchi-Beaufort (BCB) region (here defined as being bounded by 55°N-80°N and 160°E-120°W) at 2.5° grid point spacing [34] from the National Centers for Environmental Prediction/National Center for Atmospheric Research (NCEP/NCAR) were used to generate mean wind and sea level pressure fields. A directional constancy metric was adapted from *Moore* [35], here defined as the ratio of the N-day vector mean wind speed to the N-day scalar mean wind speed, to characterize winds on a variable-to-prevailing scale. Winds with directional constancy values closer to zero exhibit greater variability in their directions. Winds with values closer to one exhibit greater constancy in the direction of the mean wind (i.e. prevailing winds).

### Wind forcing and krill population structure

The relationship between ocean circulation, for which we assume broad-scale wind and sea level pressure patterns are drivers and therefore proxies for weak or strong circulation across the Chukchi Sea, and year-to-year variability in krill population structure (proportion JAD) as postulated by *Berline et al*. [11] is investigated using iterative correlation analyses (details of the methodology are in *Okkonen et al*. [8]). As employed in this study, this analytical method searches for time series of seasonally-averaged wind forcing and associated sea level pressure that vary interannually in the same manner as the time series of krill population structure. The analytical results are summarized in the form of a heat map that, in the present context, identifies the wind averaging periods (start and end dates) that maximize the aggregate ocean area north of the Bering Sea shelf break over which correlations between time series of krill life stage metrics (proportion JAD) and time-averaged winds at each NCEP grid point were statistically significant. Given the *Berline et al*. [11] result of two krill arrival events at Pt. Barrow associated with each krill brood year, an initial arrival during fall of the brood year and a second arrival the following year, our wind averaging analyses began in 2004, one year prior to our initial survey year. Spatial fields of wind vectors, wind directional constancy, statistically-significant correlations between JAD fraction and U- and V-component winds, and sea-level pressure were generated by averaging over the temporal period and spatial extent identified by the correlation analysis.

## Results

During the 11 years of the study, data from 141 off shelf ring net tows and 71 off shelf Tucker trawls (Fig 1; Table 1) were available for the analysis. Most (71–100%) of the tows were conducted during daylight (usually) or twilight. Sampling effort varied between years because of differing research objectives and weather conditions. As a result, the number of tows varied between years, ranging from 5 tows in 2006 to 17(19) ring net (Tucker) tows in 2010 (Table 1). Most tows contained krill; for a few years 1–2 tows were excluded from the calculation of ratios because krill were not present. Data from years with low numbers of available tows are included in the analysis but care is taken in interpretation of patterns based on those years.

**Fig 2:**
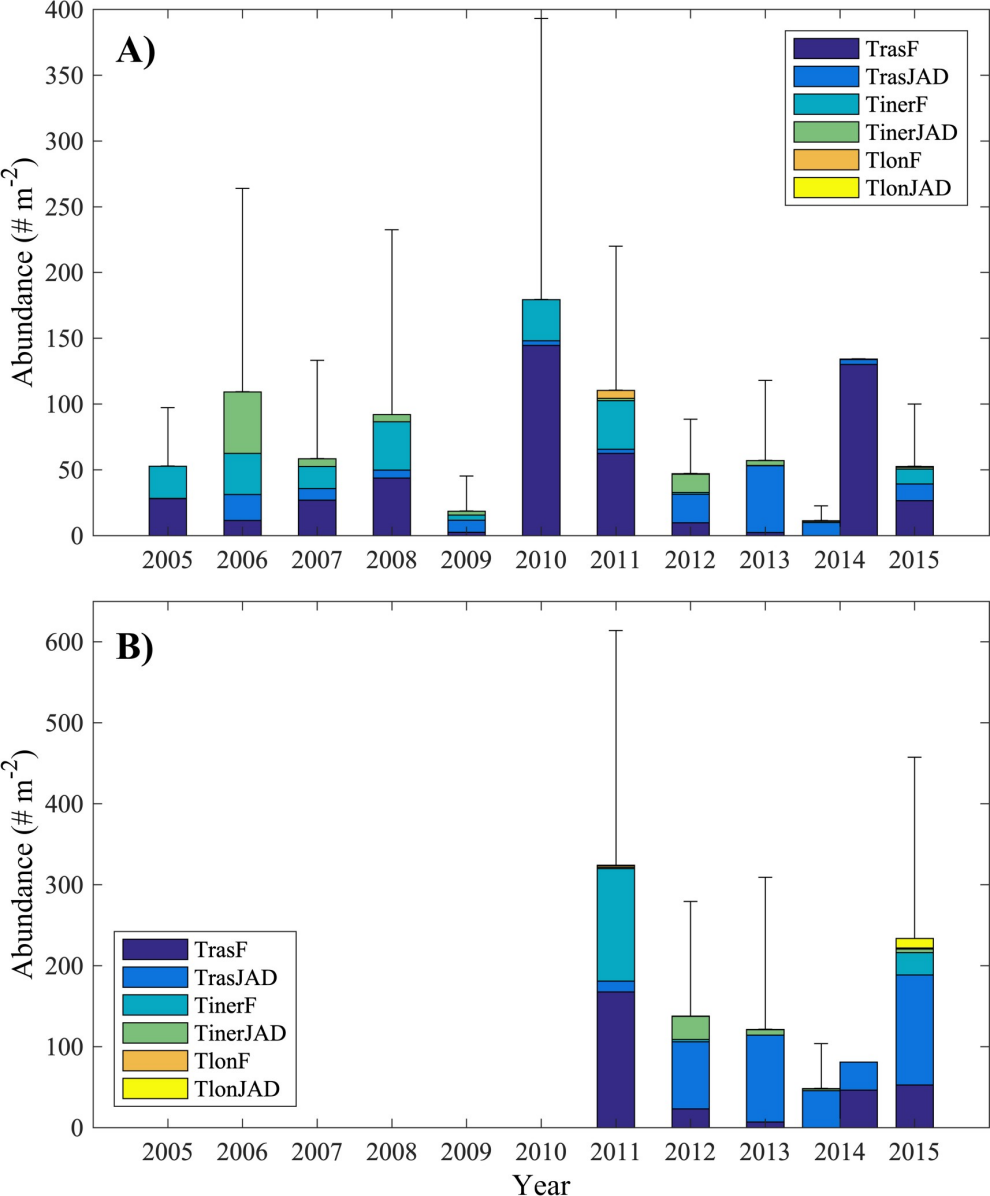
Mean annual water column abundances of Furcilia (F) and combined Juveniles and Adults (JAD) for *T*. *raschii*, *T*. *inermis*, *and T*. *longipes*. A) Abundances from samples collected using the ring net. B) Abundances from samples collected using the Tucker trawl. For 2014, data from two stations are plotted separately. Error bars show standard deviations for the total abundance. Note vertical scale difference between A) and B).

Mean abundances collected using the Tucker trawl (Fig 2B) were ~2–4 times greater than those collected using the ring net (Fig 2A). Calyptopes were present in very low abundances in some years, particularly when furcilia were the dominant life stage, however because of their low abundances were not included in this analysis. For 2014, mean abundance from two tows collected on Sept. 5 are shown separately since high abundances of furcilia, previously not present, were collected in warm, salty ACW that had appeared between that and previous sampling dates. The mean abundances collected by the ring net was ~1.5 times greater than that of the Tucker trawl for the 2014 ACW tows. Abundances of krill varied interannually and between net type for the years when both were used. Greatest ring net collected water column abundance was observed in 2010. For the period during which both nets were used (2011–2015), greatest abundances were observed in 2011 for both nets, excluding the second sampling date in 2014 that had sampled newly arrived warm, salty ACW and high abundances of furcilia. Abundances from only two stations were available for the second 2014 sampling date so the differences between the two types of gear for those mean abundances likely resulted from patchiness that was exaggerated with the small sample size. Although differences in mean total abundances between years were significant for both types of gear (p<0.05 for ring net, p<0.01 for Tucker trawl, Kruskal-Wallis test), because of the considerable variability in abundance between tows within a year (note high standard errors), this result was driven by differences between only a few years for each net (non-parametric Tukey-Kramer type post-hoc test, p<0.05; [32]). For the Tucker trawl, 2011 was significantly different from 2013 and 2014 but not from 2012 and 2015, 2014 was different from 2011 and 2015, and 2015 was different only from 2014. For the ring net, 2009 and 2011 were different and 2011 and 2014 were different, with no other significant differences between years.

**Fig 3:**
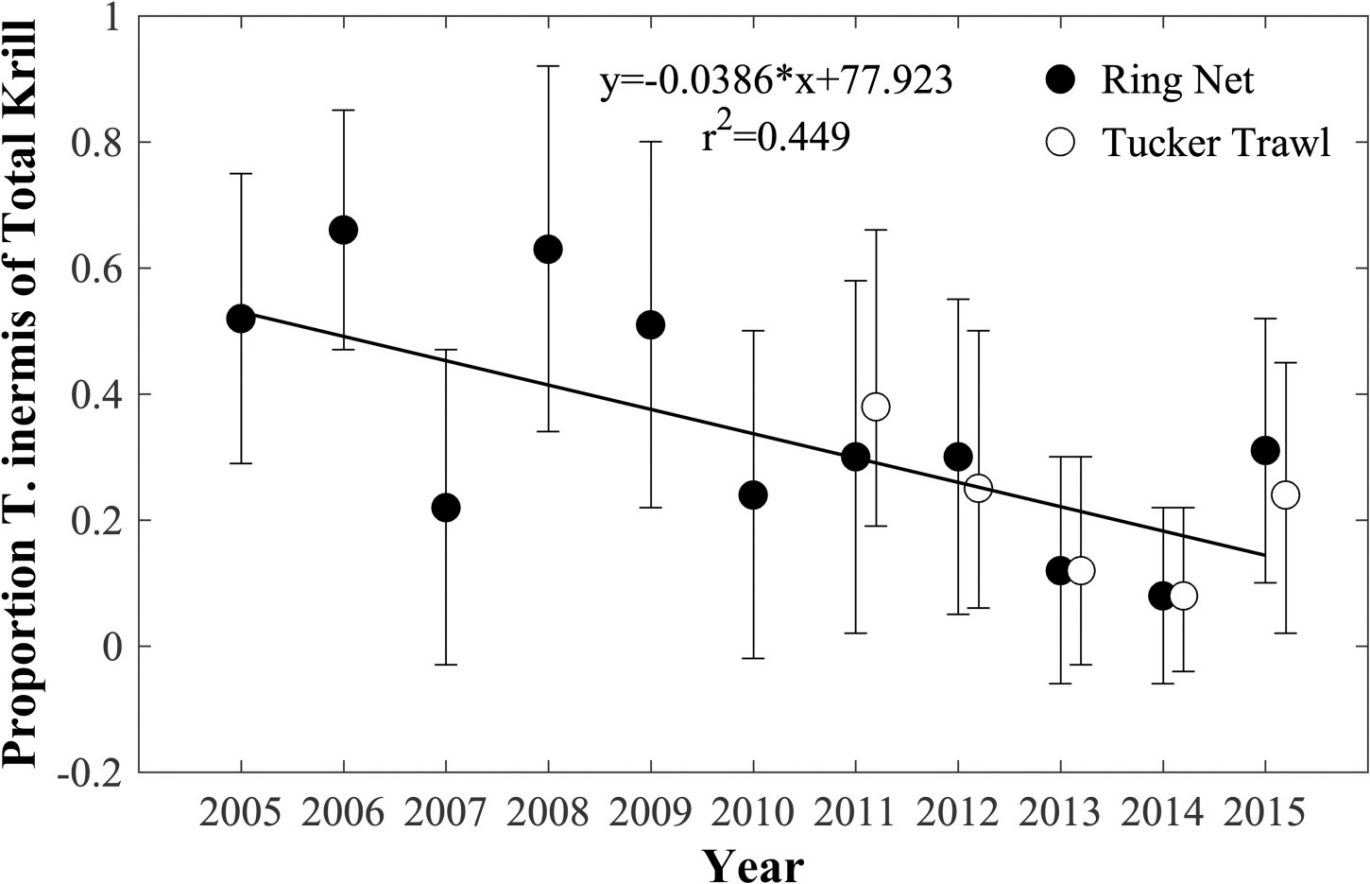
Annual mean proportion of *T*. *inermis* of the total krill abundance. Mean proportions for samples from both the ring net and Tucker trawl are shown. Regression conducted using only data from ring net. Error bars show standard deviations.

Three krill species were observed: *T*. *raschii*, *T*. *inermis*, and *T*. *longipes*. *T*. *longipes* was only infrequently observed (15 of 136 tows) in very low abundance and is not considered further. There was a gradual decline in the proportion of *T*. *inermis* of the total krill over the period (~0.04/year), with significantly less *T*. *inermis* in 2013 and 2014 than in 2005, 2006, and 2008 (Kruskal-Wallis, p<0.01; non-parametric Tukey-Kramer type post-hoc test, p<0.05) (Fig 3). The proportion of *T*. *inermis* of the total krill from the Tucker trawls was not significantly different from those of the ring net tows (Wilcoxon Paired Test).

The population structure of both krill species varied annually and together (Fig 2), with years of high proportions of juvenile and adult (JAD) *T*. *raschii* also having high proportions of JAD *T*. *inermis* (and conversely, years with high proportions of furcilia co-occurred for both species). The JAD proportions did not differ significantly between the two species within a year (t-test). Therefore, the abundances of furcilia, juveniles/adults, and total krill from the two species were combined and the proportion JAD were re-calculated from the combined abundances.

**Fig 4:**
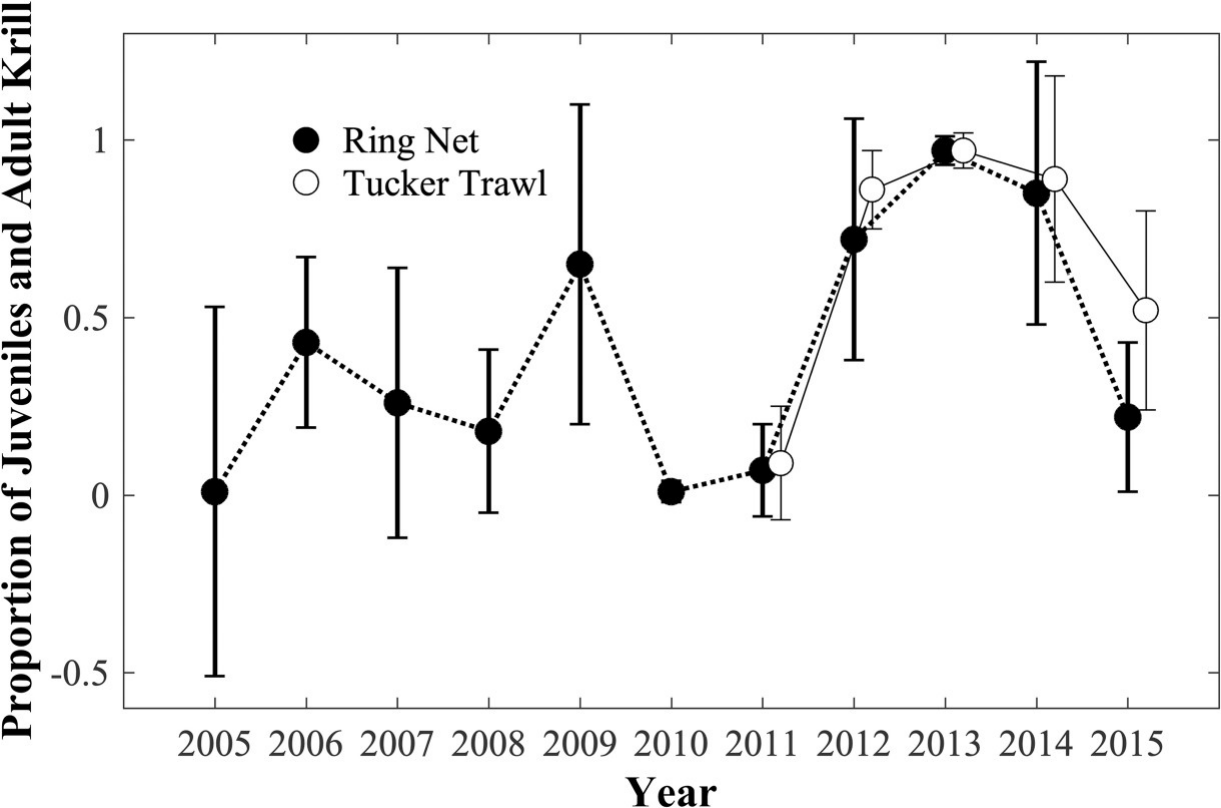
Annual mean proportion of Juvenile and Adult (JAD) krill of the total krill abundance. All species combined. Mean proportions for samples from both the ring net and Tucker trawl are shown. Error bars show standard deviations.

Significantly greater proportions of JAD were seen in 2009 and 2012–2014 (Fig 4; Kruskal-Wallis, p<0.01, non-parametric Tukey-Kramer type post-hoc test, p<0.05). The JAD proportion also was high in 2006, however only five tows were available and this difference was not significant. Almost no JAD and very high proportions of furcilia were present in 2005, 2010, and 2011 (also significantly different using same test). The proportions of JAD observed in the Tucker trawl samples were not significantly different from those of the ring net in each year with the exception of 2015 when more JAD were collected in the Tucker trawl (Mann-Whitney test, p = 0.003). Data from the two ACW samples from 2014 were included in the calculation of the mean JAD proportions for that year; had they been omitted, the proportion of JAD for both types of gear would have been 1.0. The mean proportions of JAD and integrated water column abundances were significantly negatively correlated (r = -0.66, n = 12 for ring nets, r = -0.94, n = 6 for Tucker trawls, p<0.01 for both) so that years with high mean proportions of JAD had lower mean total krill abundance.

**Fig 5:**
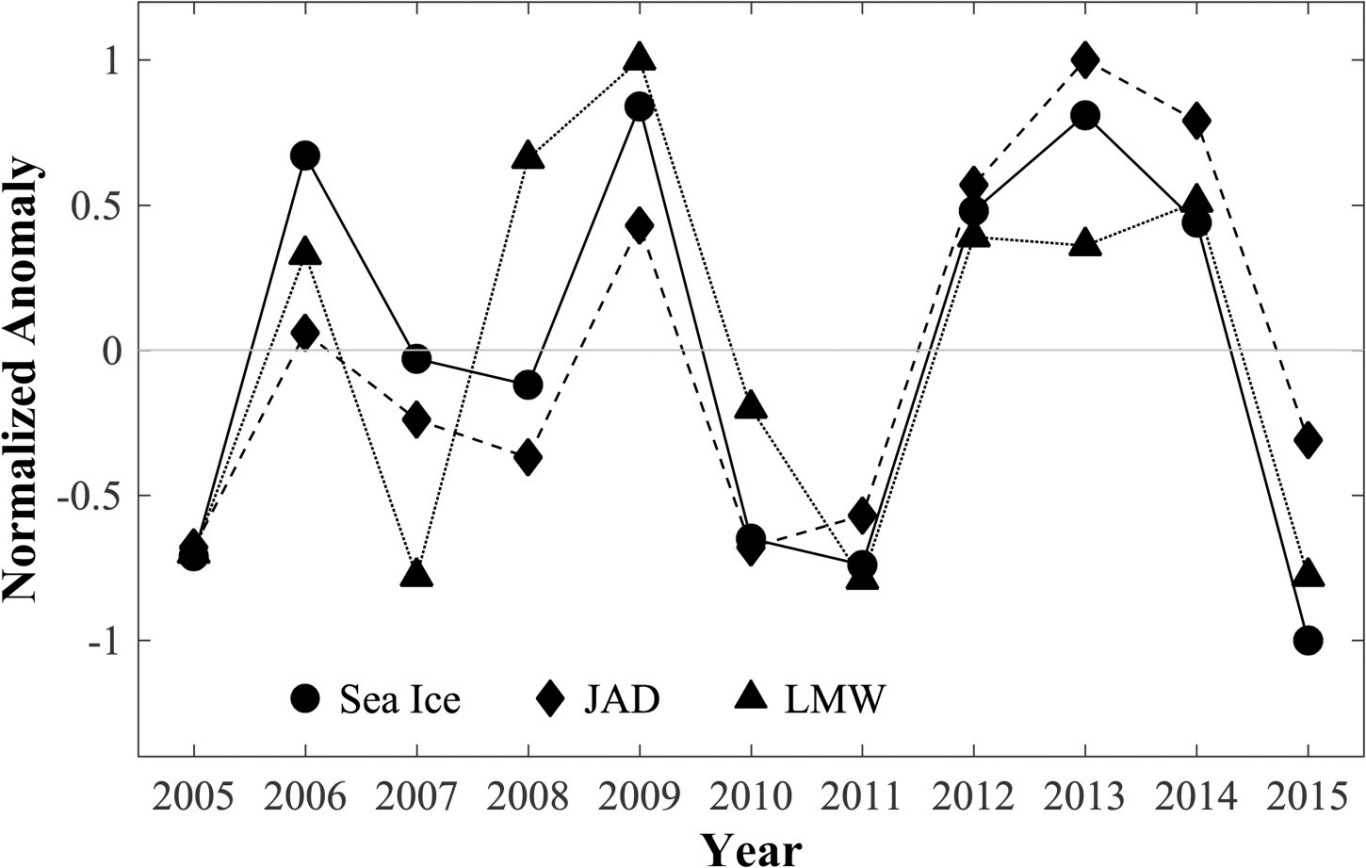
Annual normalized anomalies from the 11-year mean for May 31^st^ Chukchi Sea ice extent (sea ice), late season melt water (LMW) volume, and the proportion of combined juvenile and adult krill of total krill (JAD). LMW volume from *Okkonen et al*. [5]. Actual anomalies normalized to the maximum of the absolute values of the anomalies for each variable within the 11-year record.

Krill population structure, expressed here as the normalized anomaly of the proportions of juveniles and adults (JAD) in the populations, was significantly correlated (r = 0.85, p<0.01) with late-spring eastern Chukchi Sea ice extent (Fig 5). Krill population structure was also correlated (r = 0.66, p<0.05) with the normalized volume anomaly of LMW (Fig 5; Table 2); no significant correlations were seen between variability in the other water mass volumes (CSW, ACW, WW) and the proportions of juveniles and adults in the krill population structure. Thus, years exhibiting greater sea ice coverage in the spring (i.e. slower sea ice retreat across the Chukchi) and a greater volume of LMW in late summer were those with a high proportion of juveniles and adults in the late-summer krill population near Pt. Barrow and, conversely, a low proportion of furcilia.

**Table 2:** Summary of physical characteristics associated with either furcilia or JAD dominance in the krill population near Pt. Barrow, Alaska.

Krill Life Stage	Chukchi and Beaufort Winds	Sea Level Pressure	Sea Ice and Melt Water
Furcilia	Strong, persistent winds from E/NE during preceding fall and spring	Fall: Stronger gradient between High pressure extending from Siberia to Beaufort and Low pressure cell centered over the Alaska Peninsula. Spring: Stronger BSH in central Beaufort	Early sea ice retreat; little/no meltwater in Barrow Canyon in late summer
JAD	Weak, variable winds from E/NE during preceding fall and spring	Fall: Weaker gradient between High pressure extending from Siberia to Beaufort and Low pressure cell centered over the Alaska Peninsula. Spring: Weaker BSH in eastern Beaufort	Late sea ice retreat; more meltwater in Barrow Canyon in late summer

**Fig 6:**
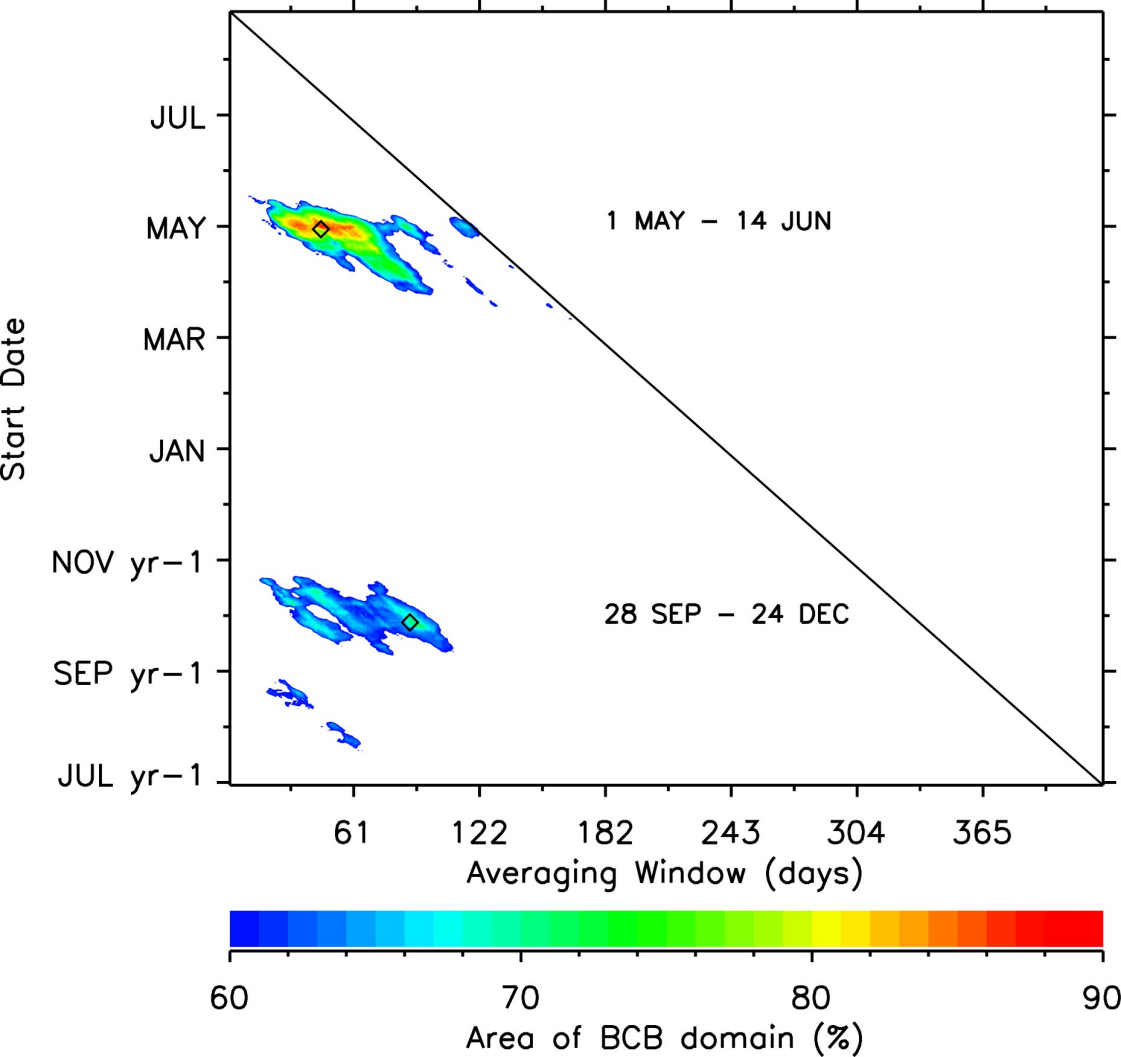
Percentage of the Bering-Chukchi-Beaufort (BCB) spatial domain with statistically significant correlations between averaged (U, V) winds and JAD fractions. Contours show % area with statistically significant correlations (r > 0.602, p<0.05; 9 degrees of freedom, two-tailed test) over a range of start dates and averaging periods. The diamond symbols identify the averaging coordinates for seasonal maxima. No results were computed for averaging period and start date pairs lying above the diagonal line because averaging periods end after 31 August of survey years.

The heat map summarizing the results of the iterative correlation analyses (Fig 6) indicates that there are two prominent wind regimes that are spatially-extensive and well-correlated with krill life stages (proportion JAD) encountered near Pt. Barrow during late summer; a fall regime and a spring regime. The greatest areal coverage for the fall wind regime (72% of the Bering-Chukchi-Beaufort (BCB) ocean area north of the Bering shelf break) is associated with an 88-day averaging period beginning 28 September in the years preceding our survey years. The greatest areal coverage for the spring wind regime (87% of the BCB ocean area) is associated with a 45-day averaging period beginning 1 May of our survey years.

**Fig 7:**
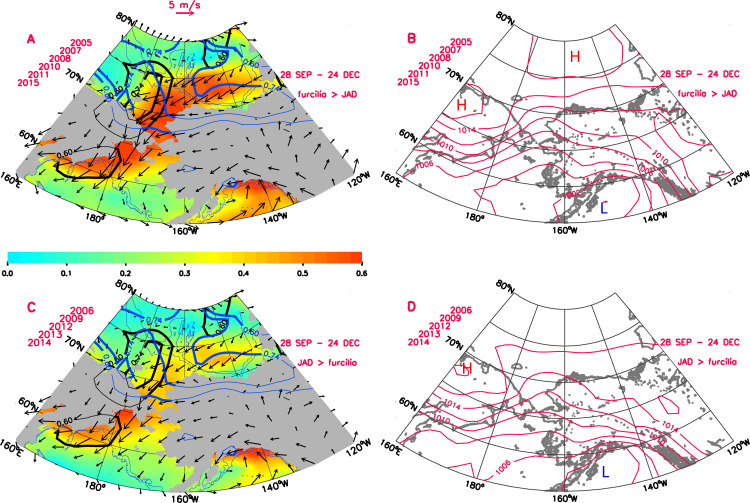
Mean atmospheric circulation from 28 September to 24 December for years preceding years in which furcilia fractions are greater (A, B) or lesser (C, D) than JAD fractions. Year annotations indicate krill survey years. A) and C) display mean wind vectors (at every 2nd i^th^ grid point), wind directional constancy (color shading) and statistically-significant correlations between JAD fraction and U-component winds (blue contours) and V-component winds (black contours) with correlation contours at r = 0.60 (p < 0.05) and 0.74 (p < 0.01). B) and D) display mean sea level pressure (hPa) patterns. High and low pressure cells are annotated with H and L, respectively. Maps generated using IDL (Interactive Data Language)/v8.6.

The mean wind and SLP fields within the Bering-Chukchi-Beaufort (BCB) region in the falls preceding the years (2005, 2007, 2008, 2010, 2011, 2015) when the furcilia fractions were greater than JAD fractions are characterized by strong and persistent (high directional constancy) northeasterly winds extending from the southern Beaufort Sea, across the Chukchi Sea to the northern Bering shelf (Fig 7A). These northeasterlies were driven by the strong pressure gradient between the Beaufort Sea High (BSH) pressure system (1017 hPa) in the central Beaufort Sea and the broad Aleutian Low pressure region (1000 hPa) over the northwestern Gulf of Alaska (Fig 7B). In contrast, in years (2006, 2009, 2012, 2013, 2014) when the JAD fraction was greater than the furcilia fraction, winds were generally weaker and more variable (low directional constancy) across most of the BCB region during the preceding-fall, although still northeasterly in the mean (Fig 7C). These weaker winds were the response to the correspondingly weaker pressure gradient between the 1017 hPa BSH and the 1003 hPa Aleutian Low (Fig 7B). While the average wind and SLP patterns depicted in Fig 7 are associated with the 28 Sep-24 Dec averaging period, they are also representative of patterns associated with other averaging periods comprising the broader fall wind regime delineated in Fig 6.

**Fig 8:**
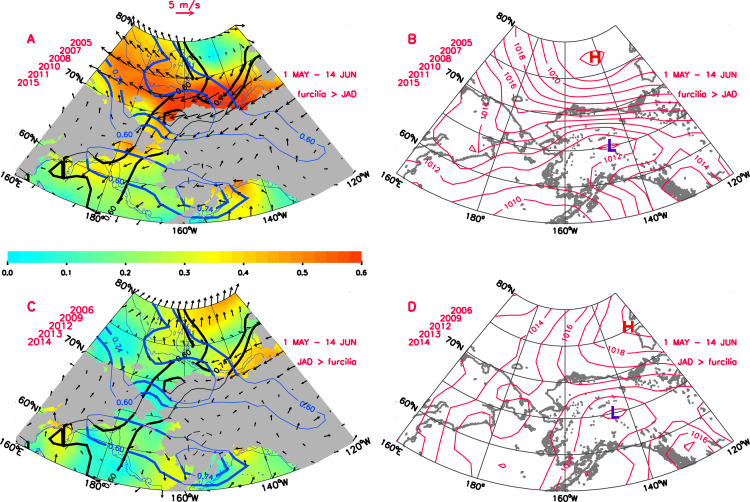
Mean atmospheric circulation from 1 May to 14 June for years in which furcilia fractions are greater (A, B) or lesser (C, D) than JAD fractions. A) and C) display mean wind vectors (at every 2nd ith grid point), wind directional constancy (color shading) and statistically-significant correlations between JAD fraction and U-component winds (blue contours) and V-component winds (black contours) with correlation contours at r = 0.60 (p < 0.05) and 0.74 (p < 0.01). B) and D) display mean sea level pressure (hPa) patterns. High and low pressure cells are annotated with H and L, respectively. Maps generated using IDL (Interactive Data Language)/v8.6.

The spring mean wind and SLP fields within the BCB region for the years when the furcilia fractions were greater than JAD fractions are also characterized by strong and persistent mostly easterly winds particularly across the southern Beaufort Sea and northern Chukchi Sea (Fig 8A). These easterlies were driven by the pressure gradient between the 1022 hPa BSH in the northcentral Beaufort Sea and 1011 hPa low pressure region over central Alaska (Fig 8C). Mean winds over the southern Chukchi and northern Bering Seas were comparatively weaker and more variable during these years.

In contrast, in years with a high JAD fraction, winds were much weaker and more variable (low directional constancy) across the southern Beaufort and northern Chukchi, although still easterly in the mean (Fig 8B). These weak winds were the response to the correspondingly weak pressure gradient between the 1019 hPa BSH in the eastern Beaufort and the 1013 hPa low pressure region over central Alaska (Fig 8D). As was the case with fall regime winds, the average wind and SLP patterns depicted in Fig 8 are also representative of patterns associated with other averaging periods comprising the broader spring wind regime delineated in Fig 6.

## Discussion

During the eleven years of this study, annual variability in late-summer krill population structure at Pt. Barrow, sea ice extent, and the volume of late season melt water (LMW) were closely correlated, patterns that could be explained by preceding fall and/or late-spring sea level pressure (SLP) patterns and wind fields over the Chukchi and Beaufort Seas (Table 2). Together with krill life history and their Bering Sea source, these associations suggested that a mechanism consisting of the interplay between wind-driven northward transport of water and sea ice, the timing of krill reproduction and development, and krill advection through the Chukchi Sea determines whether late-summer krill populations at Pt. Barrow, and in the bowhead whale feeding hotspot, consist of larger, mature krill (juveniles, adults) that overwintered in the Chukchi Sea or smaller, younger krill (furcilia) advected into the Chukchi Sea that year. Furthermore, the proportion of one of the two common krill species, *T*. *inermis*, declined from representing approximately half of the krill population at the start of the study to only about 20–30% by the end of the study.

Mean wind and SLP fields over the Chukchi Sea for the two krill life stage regimes showed very different patterns. Years when there were high proportions of juveniles and adults near Pt. Barrow in late summer (2006, 2009, 2012, 2013, 2014; Fig 5) were characterized by weaker, more variable winds over the northern Chukchi and southern Beaufort Seas the preceding-fall and in late-spring (Figs 7 and 8). The spring wind pattern also is associated with later sea ice retreat in the eastern Chukchi Sea [5], consistent with greater late spring sea ice extent seen here (Fig 5). By contrast, years when there were high proportions of furcilia near Pt. Barrow (2005, 2007, 2008, 2010, 2011, 2015) were characterized by strong, persistent easterly winds over the northern Chukchi and southern Beaufort Seas the preceding-fall and in late-spring. These years were characterized by early sea ice retreat [5] and lower late-spring sea ice extent.

How can these associations be interpreted to explain the observed interannual variability in krill population structure at Pt. Barrow? The answer lies in the interplay among atmospheric drivers, sea ice seasonal extent and annual timing of retreat, northward advection through the Chukchi, krill life history, and krill origins south of Bering Strait. The Chukchi Sea is a flow through system, with a residence time of ~5 months and a transit time from Bering Strait to the Chukchi Slope along the eastern pathway of ~100 days, based on drifters [17, 22, 36]. Therefore, the water and plankton in the Chukchi Sea turn over on at least an annual basis, flushing out to the north along the Chukchi Slope. Krill reproduce in the Bering Sea in spring and enter the Chukchi Sea through the Bering Strait as early-mid stage furcilia in late-spring or early summer, given estimated development times on the order of 1–2 months [10, 14, 15], and advance north as a furcilia front. Because krill generally are not believed to reproduce in the Chukchi Sea [10, 37], krill north of the advancing furcilia front will be more mature (JAD) and will have overwintered in the Chukchi Sea. As the summer progresses, older krill will exit the Chukchi Sea and younger krill will spread through the region. The cessation of reproduction in summer halts the supply of early stage krill to the Chukchi Sea and only older stage krill will enter the Chukchi Sea in fall or winter. By the following spring-summer, the krill overwintering in the Chukchi Sea will have developed to juvenile or even adult stages.

At Point Barrow, then, years with a high proportion of older krill in late August are those in which the furcilia front has not yet reached Pt. Barrow while years with a high proportion of younger krill are those in which the furcilia front has advanced past Pt. Barrow. The late-summer situation observed in each year depends on the characteristics of the preceding-fall and late-spring wind fields and the seasonal extent and retreat of sea ice. As demonstrated by *Okkonen et al*. [5], early sea ice retreat and low volumes of LMW are associated with a stronger BSH in late spring that occupies a more western location (i.e., north of the Chukchi Shelf) and with strong easterlies over the northern Chukchi. Later sea ice retreat and higher volumes of LMW are associated with a BSH that lies further to the east, adjacent to the Canadian archipelago, resulting in weaker winds over the northern Chukchi. Under strong easterlies, sea ice is more rapidly driven to the north, resulting in diminishing sea ice extent in the Chukchi [38–40]. Conversely, under weak winds, sea ice persists or lingers in the Chukchi Sea. Therefore, during years with strong easterly winds and early ice retreat, overwintering mature krill are flushed out of the Chukchi Sea earlier than during years with weaker winds and lingering sea ice. In late August near Pt. Barrow, mature krill that have overwintered will dominate the population during years with lingering sea ice and slower Chukchi Sea flushing when the advancing front of younger furcilia will not yet have arrived. Conversely, in years with rapid flushing, the advancing front of younger furcilia will have reached Pt. Barrow in late August and will dominate the population. The arrival of the furcilia front at Pt. Barrow in 2014 was seen in the transition between JAD dominated to furcilia dominated krill populations [Fig 2] and the arrival of much warmer ACC water on the last day of sampling in that year.

These contrasting wind conditions also influence the strength of the northward flow across the Chukchi shelf and the pathways by which water exits the Chukchi Sea. Numerical model results [41] indicate that, under strong easterly winds, northward flow through Barrow Canyon is greatly reduced and that northward flow from the Chukchi shelf into the Arctic basin is broadly distributed across the Chukchi shelf break. *Corlett and Pickart* [20] show that, under strong easterly winds, flow exiting Barrow Canyon largely turns westward, away from Pt. Barrow as the Chukchi Slope Current. Similarly, drifters deployed in July of 2011–2013 and August of 2015 in the eastern Chukchi Sea southwest of Hanna Shoal also turned westward joining the Chukchi Slope Current upon exiting Barrow Canyon [22]. Together, these observations imply that northward flow across the breadth of the Chukchi shelf break is greater in years with strong easterly winds than in years when easterly winds are weaker. The contrasting wind conditions also suggest different major pathways of krill advection through the Chukchi Sea. During strong easterly winds, more krill will be advected along the westward pathway through Herald Valley and take longer to reach Pt. Barrow while during weaker winds over the Chukchi, more krill will be advected along the Central Channel pathway and reach Pt. Barrow more quickly.

Transport through Bering Strait also varies according to the strength of the wind field [17]. Greater northward transport through Bering Strait would reduce the flushing time of the Chukchi Sea and increase the rate of northern penetration of incoming water and krill populations [17, 22]. Mean spring (May-June) transports through Bering Strait, from Fig 6 in *Woodgate* [17], were positively correlated with the proportion of furcilia in the krill population at Pt. Barrow in late August (r = +0.50, p<0.05), suggesting that in years with high spring transport, furcilia of that year reach Pt. Barrow earlier than in years with lower transport. In a broader-scale study of northeast Chukchi Sea krill distributions in August, proportions of juveniles and adults were high throughout the northeastern Chukchi in 2012 but much reduced in 2010 and 2011 [37], consistent with proportions observed in the present study at Pt. Barrow. Also in August 2012, high abundances of furcilia were observed along a transect extending to the northwest from Pt. Hope, potentially representing the advancing wave of the 2012 furcilia cohort that had not reached Pt. Barrow in late August. Thus, years with short flushing times in the Chukchi Sea result in more rapid and complete replacement of the overwintering zooplankton community with a renewed community originating in the Bering Sea.

Overwintering of krill in the Chukchi Sea has not been quantified and would be difficult to observe given that krill likely are found under and potentially in the sea ice. However, several lines of indirect evidence support the notion of an overwintering population of krill. An acoustic Doppler current profiler moored for two years (2010–2011; 2012–2013) on the western flank of Barrow Canyon recorded diel vertical migration in February-March 2011 and March-April 2013, with particularly strong diel vertical migration during March 2013, suggesting the presence of strong diel vertical migrators such as mature krill. Extensive windrows of juvenile and adult krill have been observed washed up along the shore near Utqiaġvik (formerly Barrow) Alaska in July of 2009, 2012, 2013 and 2014, consistent with the presence of a mature population of overwintering krill (C. George, pers. comm.) [28].

The causes behind the decline in the proportion of *T*. *inermis* in the total krill abundance near Pt. Barrow over the period of the study remain highly speculative. Because *T*. *inermis* are found along the outer Bering Sea shelf and *T*. *raschii* are found on the middle Bering Sea shelf, a high proportion of *T*. *raschii* might suggest that the krill reaching Pt. Barrow are originating on the middle Bering Sea Shelf and being advected north through the central or possibly eastern advective pathways. Alternatively, populations of *T*. *inermis* may have declined in the Bering Sea over the period of the study, perhaps in response to unfavorable conditions. The Bering Sea is known to alternate between warm and cold periods during which lesser or greater winter sea ice is present [42]. Preceding and during the period of this study, 2001–2005, 2014, and 2015 were “warm” years while 2007–2013 were “cold” years in the Bering Sea. It could be that *T*. *raschii* is more successful than *T*. *inermis* in cold years, resulting in the decline in the proportion of *T*. *inermis* in the Bering Sea source populations for the Chukchi Sea. The proportion of *T*. *inermis* rose slightly in 2015, coincident with the return to warm conditions.

Although interannual differences in krill abundances were observed, most were not significantly different because of the great inter-tow variability within each year likely imposed by the inherent patchiness of krill. The significantly greater Tucker collected abundances relative to the ring net abundances clearly showed the greater catch efficiency of the faster-towed Tucker trawl. Comparisons between the ring net and Tucker trawl data also demonstrated that krill population structure (proportion JAD) and species composition are independent of krill total abundance, validating the ring net proportions and providing an 11-year record of krill population structure and species composition.

A favorable feeding environment for bowhead whales, then, will depend both on the availability and size of krill, driven by krill life stage and abundance, and on mechanisms such as the krill trap [6, 7] to aggregate the krill into patches so that the whales can feed efficiently. If upwelling-favorable, easterly winds bringing krill onto the shelf do not occur during autumn, then the feeding hotspot will not form and whales will have no reason to congregate *on the shelf* near Pt. Barrow regardless of the abundance and size composition of krill in the off shelf waters. Such a situation may have occurred in 2016 when winds throughout September and October were persistently from the west (no upwelling) and in 2019 when easterly winds were too weak to drive upwelling; the result in both cases was that whales transited past Pt. Barrow quite far offshore [43, 44]. Krill abundance will depend on recruitment success in the Bering Sea, assuming that little recruitment occurs in the Chukchi Sea, on overwintering success in the Chukchi Sea, and on predation by fish and seabirds in the Bering and Chukchi Seas. Recruitment success in the Bering Sea is tied to interannual climatic variability, with warmer conditions believed to be not favorable for recruitment of larger crustaceans [45] potentially leading to lower abundances of krill supplied to the Chukchi Sea. On the other hand, environmental conditions could become favorable for krill reproduction in the Chukchi Sea, changing the paradigm outlined here. The importance of successful recruitment in the Bering Sea, predation, and overwintering in the Chukchi Sea remain important unknowns in predicting the availability of krill at the Pt. Barrow feeding hotspot.

Environmental conditions in the Chukchi Sea since 2015 have been characterized by early sea ice retreat [46], suggesting that, according to the model presented here, krill near Pt. Barrow in these years would be dominated by furcilia stages. Fall bowhead whale distributions through October 2019, observed as part of a long-term (1984-present) aerial survey effort, were notably different from previous years in that many fewer whales than normal (almost none) were found near Pt. Barrow [47]. In addition, very few whales were observed by Iñupiat hunters during the fall Utqiaġvik bowhead whale hunt (C. George, pers. comm.). Bowhead whales may have been responding to a poor feeding environment, such as small prey (furcilia), near Pt. Barrow by remaining offshore and traveling directly towards the western Chukchi Sea to intercept krill advected northwards along the Chukotka Coast.

## Conclusions

A remarkable association was observed between the life history characteristics of an important Chukchi Sea expatriate plankton taxon, euphausiids or krill, and seasonal characteristics of the physical environment, with the timing of northward transport of young-of-the year from the Bering Sea to replace the overwintering Chukchi Sea krill determining the age structure of the krill community near Pt. Barrow in late summer. The Chukchi Sea is recognized as being highly influenced, and in fact shaped, by advection of water, and intrinsic biological, physical, and chemical properties, from the north Pacific [48, 49]. Year-to-year variation in flushing through the Chukchi Sea, driven by large-scale atmospheric drivers, has a central role in the seasonal evolution of the Chukchi Sea ecosystem, in the timing of overturn of zooplankton populations in the Chukchi Sea, manifest here as the age structure of krill, and of the annual northward advancement of the furcilia front. This also determines the prey (krill) size available to bowhead whales in the northeastern Chukchi Sea. Ongoing environmental changes in the Western Arctic have the potential to disrupt multiple facets of the ecosystem, from plankton to whales and to the humans that depend on the whales for subsistence; understanding the interplay between physical drivers, Chukchi Sea flushing, and seasonal ecosystem evolution is central to predicting and understanding any climatically driven disruption.
